# Multi-dimensional attention framework for personalised Alzheimer's disease progression prediction across sporadic and genetic risk cohorts

**DOI:** 10.21203/rs.3.rs-10309496/v1

**Published:** 2026-08-03

**Authors:** Zhiyuan Song, Siyang Song, Isabel C.H. Clare, Elizabeth Head, Christy L. Hom, Adam M. Brickman, Sigan Hartley, Patrick J. Lao, Frederick A. Schmitt, Beau M. Ances, Dana L. Tudorascu, Sharon Krinsky-McHale, Florence Lai, Herminia Diana Rosas, Annie D. Cohen, Michael Rafii, Michael A. Yassa, Sid O’Bryant, Joseph H. Lee, Lauren Ptomey, Benjamin L. Handen, Bradley T. Christian, Mark Mapstone, Sarah E. Morgan, Shahid Zaman, the Alzheimer Biomarker Consortium – Down Syndrome, the Dominantly Inherited Alzheimer Network

**Affiliations:** 1 Department of Psychiatry, University of Cambridge, Cambridge, UK; 2 Department of Computer Science and Technology, University of Cambridge, Cambridge, UK; 3 School of Biomedical Engineering and Imaging Sciences, King’s College London, London, UK; 4 Department of Psychiatry, University of Pittsburgh, 3811 O'Hara St, Pittsburgh, PA, 15213, USA; 5 Waisman Center, University of Wisconsin Madison, Madison, WI, USA; 6 New York State Institute for Basic Research in Developmental Disabilities, Staten Island, NY, USA; 7 Taub Institute for Research on Alzheimer’s Disease and the Aging Brain, Columbia University, New York City, NY, USA; 8 Department of Neurology, Vagelos College of Physicians and Surgeons, Columbia University, New York City, NY, USA; 9 Washington University in St. Louis, School of Medicine, St. Louis, MO, USA; 10 Center for Neuroimaging of Aging and Neurodegenerative Disease, Massachusetts General Hospital, Boston, MA 02114, USA; 11 Department of Neurology, University of California, Irvine School of Medicine, Irvine, CA, 92617, USA; 12 Keck School of Medicine, University of Southern California, Los Angeles, CA, 90033, USA; 13 University of Kansas Medical Center, 3901 Rainbow Blvd, Kansas City, Kansas, 66160, USA; 14 Center for the Neurobiology of Learning and Memory, University of California, Irvine, CA, USA; 15 University of North Texas Health Science Center, Fort Worth, TX, USA; 16 Department of Neurology and Taub Institute for Research on Alzheimer's Disease and the Aging Brain and the Gertrude H. Sergievsky Center, Columbia University, New York, NY, 10032, USA; 17 Department of Pathology & Laboratory Medicine, University of California, Irvine School of Medicine, Irvine, California, 92617, USA; 18 Department of Neurology, University of California, Irvine School of Medicine, Irvine, California, 92617, USA; 19 Kentucky College of Osteopathic Medicine, University of Pikeville, Pikeville, Kentucky, USA; 20 Department of Neurology, University of Kentucky, Lexington, Kentucky, USA; 21 HBUG Lab, University of Exeter, EX4 4PY, Exeter, United Kingdom; 22 Alzheimer Biomarker Consortium – Down Syndrome, UK & USA; 23 Dominantly Inherited Alzheimer Network, St. Louis, MO, USA

**Keywords:** Alzheimer's disease, disease progression prediction, deep learning, attention mechanism, transfer learning, Down syndrome

## Abstract

Alzheimer's disease progresses heterogeneously across diverse cohorts, yet current predictive models fail to capture this complexity while remaining clinically interpretable. Here we present a multi-dimensional attention framework that simultaneously captures both temporal dynamics and biomarker importance to predict disease progression across three fundamentally different populations: the general late-onset population using the Alzheimer's Disease Prediction Of Longitudinal Evolution (TADPOLE) dataset (N=1669), cases with Down Syndrome-associated Alzheimer's disease using the Alzheimer's Biomarker Consortium - Down Syndrome (ABC-DS) dataset (N=396), and cases with autosomal dominant Alzheimer's disease using the Dominantly Inherited Alzheimer Network (DIAN) dataset (N=425). Trained on each dataset independently, our framework achieved multi-class Area Under the Receiver Operating Characteristic Curve (mAUC) values of 0.793 (TADPOLE), 0.680 (ABC-DS), and 0.902 (DIAN) when predicting individuals' future diagnostic status (cognitively normal/stable, mild cognitive impairment, or Alzheimer's disease) from their longitudinal biomarker history, outperforming conventional approaches. The model generates individual-specific attention maps revealing distinct biomarker importance over time. Transfer learning from TADPOLE—which included neuroimaging data—improved prediction performance on the imaging-free ABC-DS dataset from 0.680 to 0.771, demonstrating that disease mechanisms transcend both etiological boundaries and data modalities. Ultimately, this framework could enable precision medicine approaches for data-limited cohorts across the Alzheimer's disease spectrum.

## Introduction

Alzheimer's disease (AD) is a neurodegenerative disorder affecting over 55 million people worldwide ^1^. While the general population typically develops late-onset disease after age 65, genetically at-risk groups frequently experience earlier disease onset, i.e., individuals with Down syndrome (DS) show AD neuropathology by age 40 on average, with dementia onset in their mid-50s, while autosomal dominant mutation carriers develop symptoms in their 40s to 50s ^2,3^. However, disease-modifying treatments remain limited across all populations ^4^, and the disease's marked heterogeneity further complicates prognosis and treatment, emphasising the need for personalised strategies. Accurate early prediction across the disease and diagnostic spectrum (cognitively normal (CN) or cognitively stable (CS), mild cognitive impairment (MCI) and AD) could support timely, targeted intervention ^4^.

While the Alzheimer's Disease Neuroimaging Initiative (ADNI) ^5^ and similar cohorts following sporadic AD provide unprecedented multimodal longitudinal data, genetically at-risk populations, including those with DS and autosomal dominant mutations, remain understudied with smaller datasets and limited multimodal data. This disparity raises important questions about whether predictive approaches developed for well-characterised sporadic cohorts can be adapted to improve outcomes in data-limited genetic populations.

Machine learning methods have shown promise for Alzheimer's disease research, achieving high accuracy in cross-sectional diagnostic classification, although longitudinal disease progression prediction remains challenging ^6 7 8 9^. For example, studies classifying AD versus controls using vision transformers achieved pooled sensitivities (the average true positive rate across multiple test sets) exceeding 92% ^10^. However, predicting longitudinal progression is substantially more difficult due to heterogeneous progression trajectories, where disease progression rates vary substantially between individuals, with some patients declining rapidly while others remain stable for years ^6 11^. Additionally, biomarker trajectories follow nonlinear patterns where the predictive value of specific markers changes across disease stages ^6^. While prior approaches using traditional machine learning and recurrent neural networks have been applied to longitudinal AD data ^8^, they lack mechanisms to capture how biomarker importance evolves over time and to provide individual-level interpretability. Time-Aware Long Short-Term Memory networks (LSTMs) incorporate elapsed time between assessments and show high potential for other clinical prediction tasks ^12 11^, and the RETAIN model pioneered dual attention identifying influential timepoints and variables, achieving an AUC of 0.8705 for heart failure prediction ^13^. More recently, transformer-based architectures ^14^ such as the Self-supervised Transformer for Time-Series ^15^ have been adapted to irregularly sampled clinical data and applied to sporadic Alzheimer's disease cohorts. However, these attention-based and time-aware methods have to date been developed and validated within single, well-characterised cohorts, and have typically provided either temporal or feature-level interpretability rather than both simultaneously at the individual level. To our knowledge, no prior work has integrated temporal and feature-level attention within a single longitudinal framework, coupled this with individual-specific attention-map and temporal-gradient visualisations, and jointly trained, evaluated, and transferred the resulting model across genetically distinct Alzheimer's disease populations spanning sporadic late-onset, trisomic, and autosomal dominant aetiologies.

Another key challenge is that most existing approaches have not been validated across populations with different disease aetiologies, limiting understanding of whether predictive patterns generalise across genetic backgrounds ^6 7^. Although AD populations have distinct aetiologies, they share core pathophysiology ^16 17^. While the Alzheimer's Disease Prediction Of Longitudinal Evolution (TADPOLE) dataset captures late-onset sporadic disease ^18^, genetically defined populations usually follow distinct temporal patterns. More specifically, DS individuals develop early-onset AD due to chromosome 21 triplication leading to three copies of the APP genes ^19^, and autosomal dominant AD that is due to mutations in the PSEN1, PSEN2 or APP genes which also follow predictable progression ^20^. Despite different onset ages and evidence of rate of progression, the pathological sequence of AD remains consistent across populations ^16 21 22^. This conservation suggests that transfer learning, where models trained on large well-characterised datasets are adapted to improve predictions in smaller cohorts ^23^, has high potential across AD populations, revealing shared disease mechanisms while enabling model application to data-limited populations. Such an approach particularly benefits genetically at-risk populations where comprehensive data collection faces practical barriers, e.g., individuals with DS often experience anxiety during neuroimaging procedures, making extensive scanning protocols challenging ^19 24^. Meanwhile, autosomal dominant AD cohorts remain inherently small due to mutation rarity, resulting in limited sample sizes for model training ^20 25^.

Finally, achieving clinical utility from models requires not only accurate predictions but also interpretability at the individual patient level, particularly regarding how feature importance evolves over disease progression ^26^. Prior work has shown that feature importance varies substantially across disease stages ^7^, yet most existing deep learning models capture these temporal dynamics only at the population level, without providing individual-specific insights. Understanding which features drive predictions for individual patients remains a critical gap, with direct implications for personalised monitoring strategies and treatment decisions. Attention mechanisms offer a promising approach by quantifying the relative contribution of different biomarkers at each disease stage ^27^. Our framework helps address this need by generating individual-specific attention maps that reveal biomarker importance over time.

Our study addresses three key questions: (1) Can a unified attention architecture predict longitudinal AD outcomes across general and genetic populations with high accuracy? (2) Can transfer learning from general populations improve prediction accuracy for data-limited genetic cohorts? (3) What features drive predictions for different populations, how do they change over time, and are there common themes across populations? In this sense, we present a multi-dimensional attention-based deep learning framework combining temporal and feature attention to predict AD progression across TADPOLE (sporadic), ABC-DS (Down syndrome), and DIAN (autosomal dominant) cohorts. The framework provides interpretable insights while achieving state-of-the-art prediction accuracy across genetically diverse populations.

## Methods

### 1.1 Datasets

We utilised three independent cohorts to train and evaluate our multi-dimensional attention pipeline, each representing a distinct population for AD. Details of each cohort are shown in Table 1:

#### TADPOLE:

The Alzheimer's Disease Prediction Of Longitudinal Evolution (TADPOLE) dataset^18^ was drawn from the Alzheimer's Disease Neuroimaging Initiative (ADNI)^5^ and included individuals across the late-onset AD spectrum. The dataset comprised N = 1669 participants, including 502 cognitively normal controls (30.1%), 617 individuals with mild cognitive impairment (37.0%) and 550 with AD dementia (33.0%) at the baseline study visit. The data collected included a comprehensive set of biomarkers—neuroimaging (MRI and PET), cerebrospinal fluid (CSF) analytes, cognitive assessments and demographic variables, measured over multiple clinical visits (mean 6.7 visits per participant).

#### ABC-DS:

The Alzheimer's Biomarker Consortium - Down Syndrome study ^19^ focused on individuals with DS who are at high risk for early-onset AD due to trisomy 21. The cohort included N = 396 participants with DS, consisting of N = 327 cognitively stable individuals (82.6%), N = 29 with mild cognitive impairment (7.3%), and N = 40 with AD dementia (10.1%) at the baseline study visit. The longitudinal data (mean 2.8 visits per participant) collected included detailed cognitive tests, blood-based biomarkers, and demographic information. We did not have access to neuroimaging measures for this cohort.

#### DIAN:

The Dominantly Inherited Alzheimer Network dataset ^25^ comprised participants carrying autosomal dominant mutations in PSEN1, PSEN2, or APP that lead to familial early-onset AD. This dataset contained N = 425 individuals from families with autosomal dominant AD mutations, including N = 304 cognitively normal participants (71.5%), N = 45 with mild cognitive impairment (10.6%) and N = 76 with AD dementia (17.9%) at the baseline study visit. This cohort included multimodal biomarkers similar to TADPOLE (neuroimaging, fluid biomarkers, cognitive assessments) but in a genetically defined population with a virtually certain disease trajectory ^25^ (mean 3.2 visits per participant).

### 1.2 Experimental Setup and Evaluation

We trained and assessed our model on each of the three datasets independently to evaluate performance across diverse AD populations. For each dataset, we employed a stratified data splitting strategy to ensure robust and unbiased assessment. We first created a hold-out test set comprising 10% of the participants from each individual dataset, stratified by age, number of clinical visits, gender, and diagnostic status (CN/CS, MCI and AD), to ensure the test set contained proportionally representative samples compared to the original dataset (see **Supplementary Figures 1, 2 and 3**). The remaining 90% of each dataset was used for model training with 5-fold cross-validation, which helped mitigate potential selection bias and overfitting.

We used all longitudinal data up to each participant's second-to-last visit to generate month-by-month diagnostic predictions for the subsequent 10-year period. Each participant's actual diagnosis at their final visit, which occurred at varying points within this window, served as a ground truth to validate the predicted trajectory. This approach evaluated the model's ability to forecast disease progression across diverse temporal horizons, as participants’ final visits occurred at different intervals from baseline.

### 1.3 Data Preprocessing and Organisation

#### 1.3.1 Missing Data Imputation

A significant challenge in making predictions from longitudinal Alzheimer's data is the prevalence of missing values, both within individual clinical visits and across the temporal sequence. We employed a K-Nearest Neighbours (KNN) imputation strategy to address this challenge ^28^. For baseline clinical visits, we implemented KNN imputation to ensure all participants had complete initial data prior to longitudinal modelling. We identified the K most similar participants based on available observed biomarkers and utilised their measurements to estimate missing biomarkers for each individual. The similarity between participants was calculated using Euclidean distance in the feature space of available measurements, with K determined through cross-validation on the training data to optimise imputation accuracy while maintaining computational efficiency ^28^. For subsequent clinical visits, we applied the same KNN methodology with temporal considerations, where the imputation model incorporated both the cross-sectional information (similarities between participants computed using all available data from baseline through the current timepoint) and longitudinal patterns from the participant's own measurement history from prior timepoints. To prevent information leakage, all imputation parameters were derived exclusively from the training partition within each cross-validation fold. The pool of candidate neighbours and the value of K were determined using training data only; validation and held-out test participants were never used as neighbours for one another, and their missing values were imputed solely from training-set participants. The 10% hold-out test set was imputed only after the imputation model had been fixed on the training data.

After identifying the K nearest neighbours for a participant with missing values, data imputation was performed using a standard weighted average, where the contribution of each neighbour was inversely proportional to their distance from the target participant:

$${\hat{x}}_{i,f}=\frac{\sum_{j\in N_{K}(i)}x_{j,f}\cdot w(i,j)}{\sum_{j\in N_{K}(i)}w(i,j)}$$

where ${\hat{x}}_{i,f}$ is the imputed value for feature f in participant *i*, *N_K_*(*i*) represents the set of K nearest neighbours for participant *i*, and *w*(*i, j*) is the weight assigned to neighbour *j*, calculated as *w*(*i, j*) = 1/(*d*(*i, j*) + *ε*), with *ε* = 10^−8^ to prevent division by zero when a neighbour has identical feature values to the target participant. This approach ensures that more similar participants contribute more significantly to the imputed values.

#### 1.3.2 Feature Scaling and Normalisation

Before model training, all imputed datasets underwent feature scaling and Normalisation to standardise ranges across heterogeneous biomarkers. Each feature was normalised by subtracting the mean of that feature across participants and dividing by the standard deviation across participants calculated from the training data:

$$x_{i,f}^{\prime }=\frac{x_{i,f}-\mu_{f}}{\sigma_{f}}$$

where $x_{i,f}^{\prime }$ is the normalised value, and $\mu_{f}$ and $\sigma_{f}$ are the mean and standard deviation of feature f across participants in the training data, respectively.

### 1.4 Time-Aware Feature Attention Mechanism

**Figure 1:**
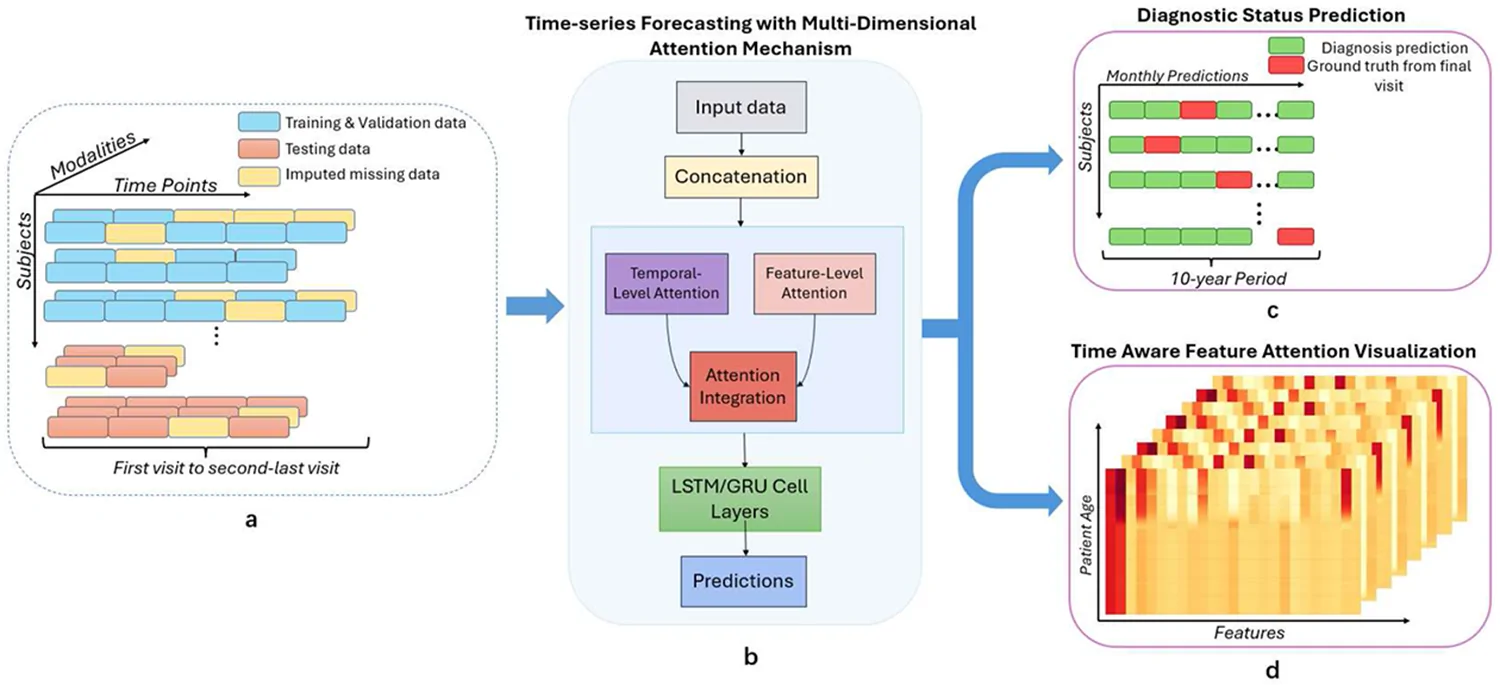
Overview of the proposed multi-dimensional attention framework for Alzheimer's disease progression prediction. (a) Multi-modal longitudinal dataset structure. Each participant has varying numbers of clinical visits over time, with different biomarker modalities collected at each visit. Stratified division into training/validation (blue) and testing (orange) datasets, with missing data imputation (yellow) applied to complete the longitudinal sequences. (b) Multi-dimensional attention framework architecture. The pipeline shows the data flow through temporal attention, feature attention, attention integration and recurrent neural layers (LSTM/GRU) to predict diagnostic status for AD progression. (c) Disease progression forecasting. The model generates month-by-month diagnostic predictions for a 10-year period following each participant's second-to-last visit. The actual diagnosis at the final visit, occurring at any point within this 10-year window, serves as ground truth for validating the predicted trajectory. (d) Time-aware feature attention visualisation. The model generates individual-specific attention maps revealing biomarker importance across time.

The core innovation of our framework is a multi-dimensional attention mechanism that simultaneously captures both temporal and feature-level importance. This mechanism, illustrated in Figure 1, comprises three integrated components that work together to identify the most relevant clinical information across both time and measurement domains.

#### Temporal-Level Attention:

This component weighs the importance of different time points in a participant's clinical history, addressing the fundamental challenge that not all visits carry equal predictive value for disease progression. The temporal attention module incorporates elapsed calendar time between visits as a continuous variable, recognising that the significance of measurements may decay or evolve non-linearly over time. Mathematically, this temporal weighting is expressed as:

$$\alpha^{t}=softmax(f_{t}([h_{t},\Delta t]))$$

where *h_t_* represents the hidden state at time *t*, *Δt* represents the time elapsed since the previous measurement and $f_{t}$ is a learned non-linear transformation function implemented as a two-layer neural network with Rectified Linear Unit (ReLU) activation ^29^. We employed the SoftMax activation function for temporal attention to create a normalised probability distribution across clinical visits, ensuring the model focused on the most informative visits while maintaining interpretability ^30^. This normalised attention distribution enabled the model to identify which clinical visits carried the most prognostic value.

#### Feature-Level Attention:

This component employed a shared attention mechanism that was learned from all participants during training to identify general patterns of biomarker importance across disease stages. When applied to individual participants, these learned parameters interacted with individual-specific biomarker values to generate personalised attention weights, ensuring that feature importance adapted to each participant's unique clinical profile while benefiting from population-level insights ^31^. The feature attention module employed a learned transformation that mapped input features to attention weights:

$$\alpha^{f}=tanh(W_{f}\cdot x+b_{f})⊙w_{f}$$

where $W_{f}$ and $b_{f}$ are learnable parameters, *x* is the feature vector, $w_{f}$ is a feature weight vector, and ⊙ represents element-wise multiplication. We utilised the hyperbolic tangent (*tanh*) activation function for feature attention, allowing the model to both emphasise relevant features and suppress misleading ones through its bidirectional output range ^32^. Unlike SoftMax, tanh preserves the independent contribution of multiple biomarkers, which is an essential property for AD where parallel pathological processes often manifest simultaneously.

#### Attention Integration:

The temporal and feature attention mechanisms were integrated through a layer Normalisation process ^33^ that ensured stable training dynamics. The integrated attention weights were applied to the input before being processed by the recurrent neural network layers:

$$\hat{x}=LayerNorm(x⊙\alpha^{f})⊙\alpha^{t}$$

where $x\in R^{d}$ represents the input feature vector at a given time step with *d* dimensions corresponding to different biomarkers, $\alpha^{f}\in R^{d}$ represents the feature attention weights computed through the tanh-based attention mechanism, $\alpha^{t}\in R$ represents the temporal attention weight for the current time step computed through SoftMax Normalisation, *LayerNorm* denotes the layer Normalisation operation that standardises activations across the feature dimension and $\hat{x}\in R^{d}$ represents the final attention-weighted feature vector. This integration mechanism sequentially applies feature-level attention to identify important biomarkers, normalizes the resulting values to maintain stable gradients, and then applies temporal attention to weight the contribution of different time points in the participant's disease trajectory ^34 35^.

### 1.5 Recurrent Neural Network Architecture

The attention-weighted features from our multi-dimensional attention mechanism were processed through a recurrent neural network that modelled the temporal evolution of biomarkers. We selected recurrent neural networks over alternatives such as transformers primarily due to the variable-length nature of participant data in our datasets. RNNs naturally accommodated this variability without requiring padding or truncation, processing each participant sequence according to its actual length ^36.^

### 1.6 Implementation Details

Our framework supported multiple RNN variants, including Long Short-Term Memory (LSTM) ^12^ and Gated Recurrent Unit (GRU) ^37^. These gated RNNs selectively remembered long-term dependencies (such as baseline measurements that remain predictive years later) while adaptively incorporating new information when disease state changes occur ^11^. The architecture employed dropout regularization applied separately to input and hidden states, multiple stacked RNN layers to capture hierarchical temporal patterns, and residual connections to maintain access to earlier time point information. Our RNN implementation explicitly accounted for irregular intervals between visits by accepting time difference information (*Δt*) as an additional input, allowing the model to adjust its state updates according to the actual elapsed time between measurements.

### 1.7 Transfer Learning and Adaptation

We used transfer learning to leverage the comprehensive biomarker patterns learned from the TADPOLE dataset when adapting to more specialised and data-limited populations represented by ABC-DS and DIAN ^38^. Transfer learning selectively preserved knowledge in specific components of our neural architecture ^39^. We froze the feature attention mechanism and first recurrent layer, as these components captured fundamental disease progression patterns and biomarker relationships. Meanwhile, subsequent recurrent layers and output projection layers remained trainable, allowing adaptation to population-specific characteristics.

### 1.8 Training Process and Regularization

The model was trained using an early stopping strategy ^40 41^ with patience and minimum improvement thresholds to prevent overfitting while ensuring optimal performance. Specifically, we monitored the cross-entropy loss on validation data during training and halted the process when no improvement exceeding a predefined threshold (0.001) was observed for 10 consecutive epochs ^40^. This approach automatically determined the optimal training duration for each dataset. After validation performance plateaued, we saved the model weights that achieved the best validation performance. The training process employed cross-entropy loss for diagnostic classification. We implemented learning rate scheduling with step decay to improve convergence, gradually reducing the learning rate when performance plateaued. The learning rate was initialised at 0.001 and reduced by a factor of 0.3 every 5 epochs when validation performance showed no improvement ^42^. We also applied an L2 regularization term with a coefficient of 10^−5^ to all model parameters. This regularization approach prevented individual weights from becoming excessively large, effectively constraining the model's complexity and reducing the risk of overfitting to training data peculiarities that might not generalise to new populations ^43^.

### 1.9 Baseline Models for Comparison

We benchmarked our framework against a range of baselines spanning classical and deep learning approaches. Non-sequential baselines comprised a Support Vector Machine (SVM) and eXtreme Gradient Boosting (XGBoost) ^44^ applied to concatenated longitudinal features. Sequential baselines without attention comprised standard GRU and LSTM networks. Two transformer-based architectures were also evaluated, namely a standard Transformer encoder and the Self-supervised Transformer for Time-Series (STraTS), which is designed for irregularly sampled multivariate time series. For ABC-DS and DIAN, we additionally evaluated TADPOLE-pretrained variants of the Transformer, STraTS, and our proposed model to determine whether transfer learning benefits extend beyond our architecture. All baselines were trained under identical data splits and preprocessing pipelines as our proposed framework.

### 1.10 Evaluation Metrics

The primary evaluation metric we employed was the multi-class Area Under the Receiver Operating Characteristic Curve (mAUC), to assess how accurately our model predicted a participant's future diagnostic state (CN, MCI, or AD) based on their longitudinal biomarker history, in a way that was robust to class imbalance across the three diagnostic categories.

The mAUC was calculated by averaging the AUC values for all possible pairwise class comparisons:

$$mAUC=\frac{2}{c(c-1)}\sum_{i<j}\frac{\hat{A}(i\;|\;j)+\hat{A}(j\;|\;i)}{2}$$

Where $\hat{A}(i\;|\;j)$ represents the probability that a randomly drawn member of class *j* will have a lower estimated probability of belonging to class i than a randomly drawn member of class *i*, and c is the total number of classes. This formulation ensured that the metric performed consistently regardless of class imbalance in the dataset and did not require specification of classification thresholds or misclassification costs.

### 1.11 Ethics approval and consent to participate

Ethical approval for the present study was provided under UK Health Research Authority (HRA) and Health and Care Research Wales (HCRW) Approval, granted on 2 May 2023, following a favourable opinion from the North West – Greater Manchester South Research Ethics Committee (REC reference: 23/NW/0002; IRAS project ID: 287116; favourable opinion dated 27 January 2023). The study was sponsored by Cambridgeshire and Peterborough NHS Foundation Trust and the University of Cambridge. All analyses were performed in accordance with the Declaration of Helsinki and relevant guidelines and regulations.

This study analysed de-identified data from three source studies: the Alzheimer's Disease Neuroimaging Initiative (ADNI), the Alzheimer's Biomarker Consortium–Down Syndrome (ABC-DS), and the Dominantly Inherited Alzheimer Network (DIAN). Each source study was approved by its respective institutional review boards — ADNI by the institutional review boards of all participating sites; ABC-DS by the University of Pittsburgh Institutional Review Board and local IRBs at each participating site; and DIAN by the Washington University in St. Louis Institutional Review Board and local IRBs/IECs at each participating site. All participants, or their legally authorised representatives, provided written informed consent for their de-identified data to be used in research.

## Results

### 2.1 Performance Comparison

Table 2 presents the models’ performance across the TADPOLE, ABC-DS and DIAN datasets.

For the TADPOLE dataset, the multi-dimensional attention GRU achieved mAUC values of 0.856 (validation) and 0.793 (test), outperforming the SVM (0.682/0.665), XGBoost (0.732/0.709), GRU (0.771/0.677), LSTM (0.764/0.685), Transformer (0.795/0.754), and STraTS (0.851/0.751). Although STraTS achieved a comparable validation mAUC, its validation-test gap of 0.100 exceeded the 0.063 gap of our proposed model, indicating greater overfitting. The validation-test gap for our model (0.063) also compared favourably with GRU (0.094) and LSTM (0.079), suggesting better generalisation to unseen participants.

The same performance pattern was observed in the ABC-DS and DIAN cohorts. In ABC-DS (without transfer learning), the multi-dimensional attention GRU achieved 0.642/0.680, exceeding the GRU (0.629/0.637), LSTM (0.618/0.651), and XGBoost (0.654/0.671). Notably, both the Transformer (0.585/0.543) and STraTS (0.547/0.553) underperformed the SVM (0.583/0.613), suggesting that transformer architectures may struggle with the limited sample size and reduced biomarker availability of ABC-DS. For DIAN, our model reached 0.814/0.902, surpassing XGBoost (0.788/0.767), GRU (0.783/0.767), LSTM (0.779/0.767), Transformer (0.796/0.762), and STraTS (0.714/0.735). Across all three datasets, the multi-dimensional attention GRU consistently achieved the highest test mAUC.

### 2.2 Transfer Learning from TADPOLE Dataset

Transfer learning from TADPOLE improved the ABC-DS validation performance by 21.2% (from 0.642 to 0.779) and test performance by 13.4% (from 0.680 to 0.771). We note that the ABC-DS dataset did not include the neuroimaging features present in TADPOLE, suggesting that pre-training on neuroimaging data can improve prediction accuracy on data where no neuroimaging is available, likely due to disease mechanisms that transcend aetiological boundaries and data modalities. Transfer learning also improved the transformer-based baselines on ABC-DS (Transformer: 0.585/0.543 to 0.689/0.665; STraTS: 0.547/0.553 to 0.715/0.666), yet neither matched our TADPOLE-pretrained model (test mAUC 0.771).

The prediction metrics in the DIAN dataset showed minimal change with transfer learning, with validation performance decreasing by 0.004 and test performance remaining unchanged at 0.902. This suggests that populations with deterministic mutations and relatively comprehensive multimodal data may not benefit from knowledge transfer from sporadic AD cohorts.

### 2.3 Stratified Performance Analysis

Stratified analysis of our multi-dimensional attention GRU model on the TADPOLE cohort revealed how participant characteristics influenced prediction accuracy. The strongest predictor was the number of longitudinal assessment points: participants with six or more visits achieved an average mAUC of 0.917, compared to 0.826 for 4-5 visits and 0.808 for 2-3 visits, confirming that temporal attention mechanisms benefit from richer longitudinal information as expected. Age showed an inverse relationship with mean accuracy, declining from 0.876 in participants under 65 to 0.826 (ages 65-75) and 0.813 (over 75), likely reflecting the challenge of distinguishing pathological decline from normal ageing-related changes in older participants.

The interval between the penultimate and final assessments had limited impact on prediction accuracy (0-6 months: 0.803; 6-18 months: 0.821; >18 months: 0.786), suggesting the model maintains robustness across different prediction horizons. Similarly, demographic factors showed negligible effects: the model achieved nearly identical performance on female and male participants (0.820 vs 0.819). While the TADPOLE cohort lacks substantial racial diversity (predominantly White at 83.8%), analysis of the available data showed limited performance variation across groups: Asian (mAUC = 0.831, N = 78), More than one race (mAUC = 0.824, N = 71), White (mAUC = 0.820, N = 1394), apart from somewhat reduced accuracy for Black participants (mAUC = 0.787, N = 118). These findings suggest the model mostly captures disease patterns rather than demographic confounds, supporting its generalisability to the heterogeneous patient populations encountered in routine clinical practice, although further work in more diverse samples including greater numbers of Black participants will be important.

### 2.4 Feature Attention Analysis – TADPOLE

**Figure 2:**
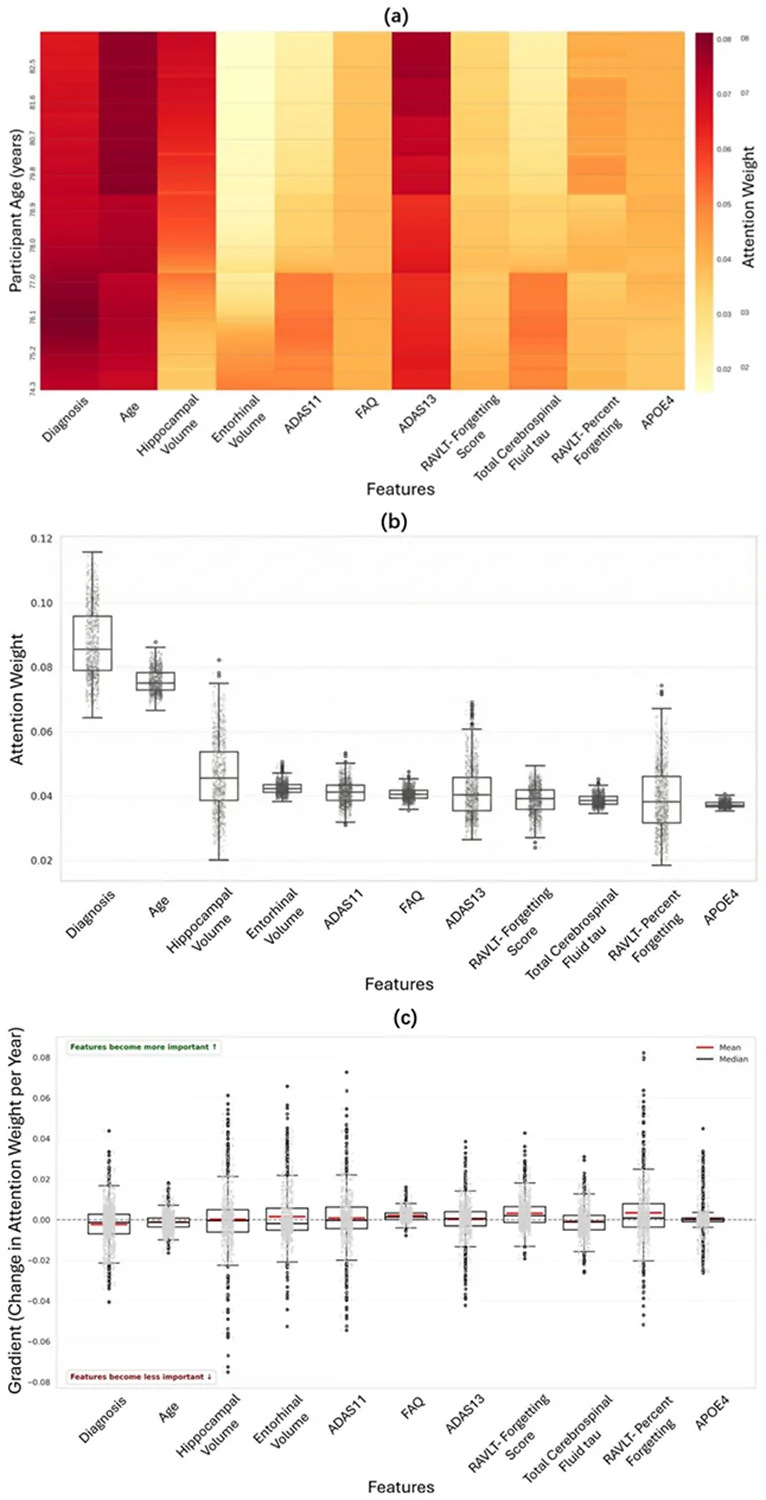
Feature attention analysis for the TADPOLE cohort. (a) Individual-Specific Attention Patterns of a TADPOLE Participant: In this example, an individual participant's attention map showed high personalised weights for diagnosis and age throughout their specific disease trajectory from age 74-83. Cognitive measures (Alzheimer's Disease Assessment Scale-Cognitive Subscale 13 items (ADAS13)) and structural markers (Hippocampal Volume) showed increasing importance with age. The participant was diagnosed as MCI at age 81. **(b) Distribution of Personalised Feature Attention Weights Across TADPOLE Cohort:** Diagnosis and age dominated with highest attention weights, while cognitive (Alzheimer's Disease Assessment Scale-Cognitive Subscale 11 items (ADAS11), ADAS13, Functional Activities Questionnaire (FAQ), Rey Auditory Verbal Learning Test (RAVLT)) and structural (Hippocampal and Entorhinal Volume) markers showed moderate importance with greater inter-participant variability. APOE4 demonstrated lowest but consistent attention across participants, aligning with its role in disease initiation rather than progression monitoring. **(c) Temporal Gradients of Feature Importance Across Disease Progression of TADPOLE Cohort:** Feature importance shifted from molecular markers (Total Cerebrospinal Fluid tau) to cognitive-functional measures (RAVLT, FAQ) and structural markers (Entorhinal Volume) as Alzheimer's disease progressed, capturing the evolution from biochemical changes to clinical symptoms.

Figure 2a shows a personalised attention heatmap for a representative participant from the TADPOLE dataset, demonstrating how feature importance evolves uniquely across their individual disease trajectory from age 74 to 83. We computed the distribution of personalised feature attention weights for the top 10 features plus APOE4 across all TADPOLE participants, see Figure 2b. We observed substantial heterogeneity in attention patterns, with hippocampal volume and cognitive measures showing wide variances, suggesting diverse progression phenotypes even within sporadic AD. Diagnosis and age demonstrated the highest median attention values (0.086 and 0.077 respectively) with relatively tight distributions (coefficient of variation < 0.20), confirming their role as anchoring features across all participants.

Finally, Figure 2c presents the temporal gradients of feature importance, revealing a fundamental shift in biomarker relevance during AD progression. Gradients were calculated as the change in attention weight for each year of follow-up, capturing whether a feature’s importance increased or decreased over time. The gradient analysis uncovered a critical transition from molecular to clinical markers as the disease advances. Notably, the total cerebrospinal fluid tau biomarker exhibited a negative gradient (reduced feature importance over time), potentially in-line with recent evidence that CSF total tau biomarkers can plateau in advanced disease stages due to extensive neuronal loss reducing the available substrate for tau release ^45 46^, and longitudinal studies showing tau accumulation rates diminish as available binding sites become saturated ^45 47^. Conversely, cognitive and functional measures demonstrated positive gradients, indicating their importance increased over time. The Rey Auditory Verbal Learning Test score (a measure of memory consolidation failure), the Alzheimer's Disease Assessment Scale-Cognitive Subscale (a measure of cognitive functional decline), and the Functional Activities Questionnaire (a measure of instrumental activities) showed stage-dependent predictive importance. Memory measures peaked in early stages, while functional measures became critical in later progression. This pattern captured the biological reality that as molecular pathology plateaus, clinical manifestations become the primary drivers of diagnostic transitions. The model successfully identified this shift from upstream molecular processes to downstream clinical consequences.

### 2.5 Feature Attention Analysis – ABC-DS and DIAN

**Figure 3:**
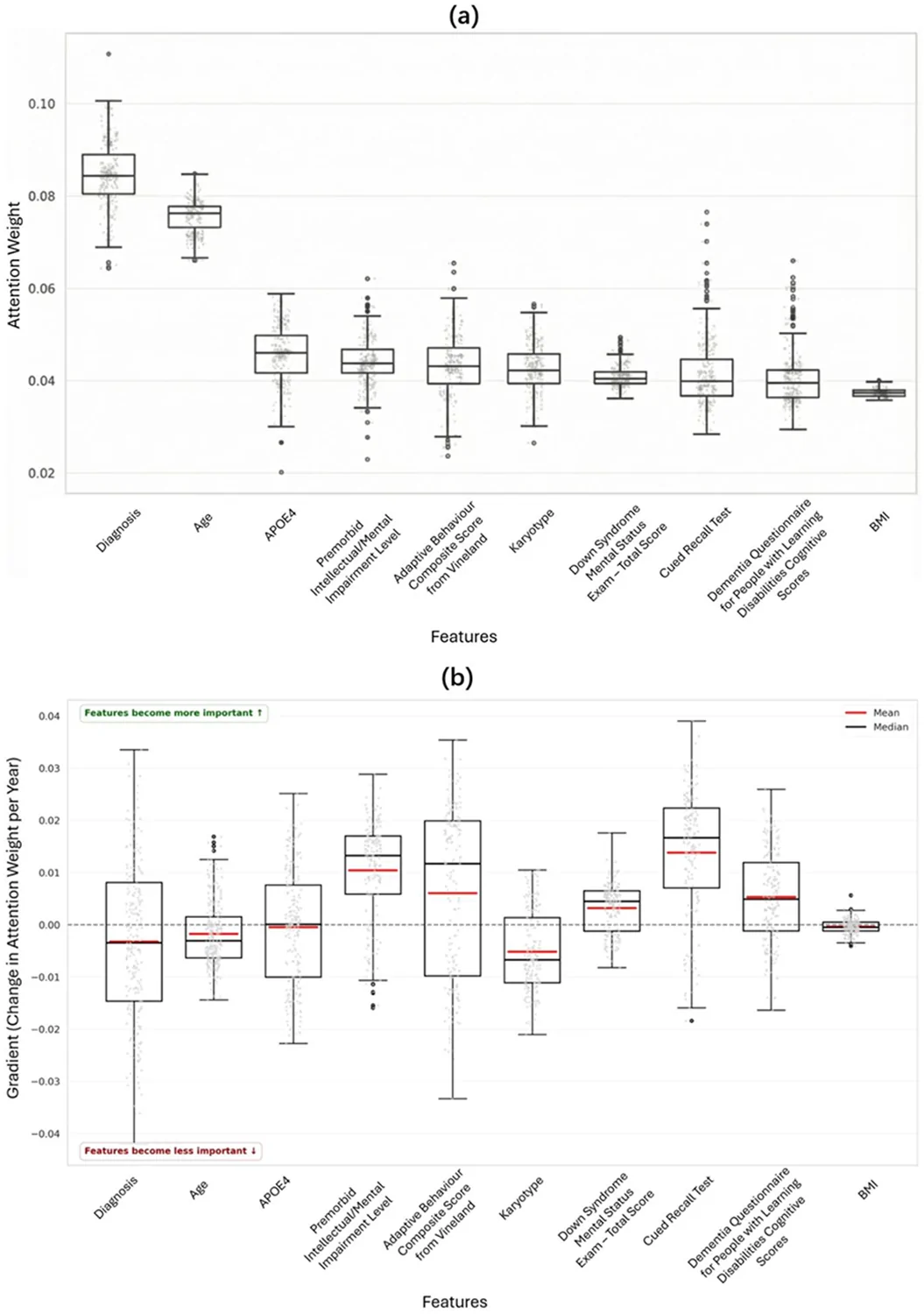
Feature attention analysis for the ABC-DS cohort. **(a) Distribution of Personalised Feature Attention Weights Across ABC-DS Cohort:** APOE4 showed high median attention across participants. Memory scores, functional measures and Karyotype received elevated attention weights. **(b) Temporal Gradients of Feature Importance Across Disease Progression of ABC-DS Cohort:** Memory and functional measures (Cued Recall Test, Premorbid Intellectual/Mental Impairment Level, and Adaptive Behaviour Composite Score from Vineland) displayed the strongest positive gradients, while Karyotype exhibited the most negative gradient, followed by Age and Diagnosis.

**Figure 4:**
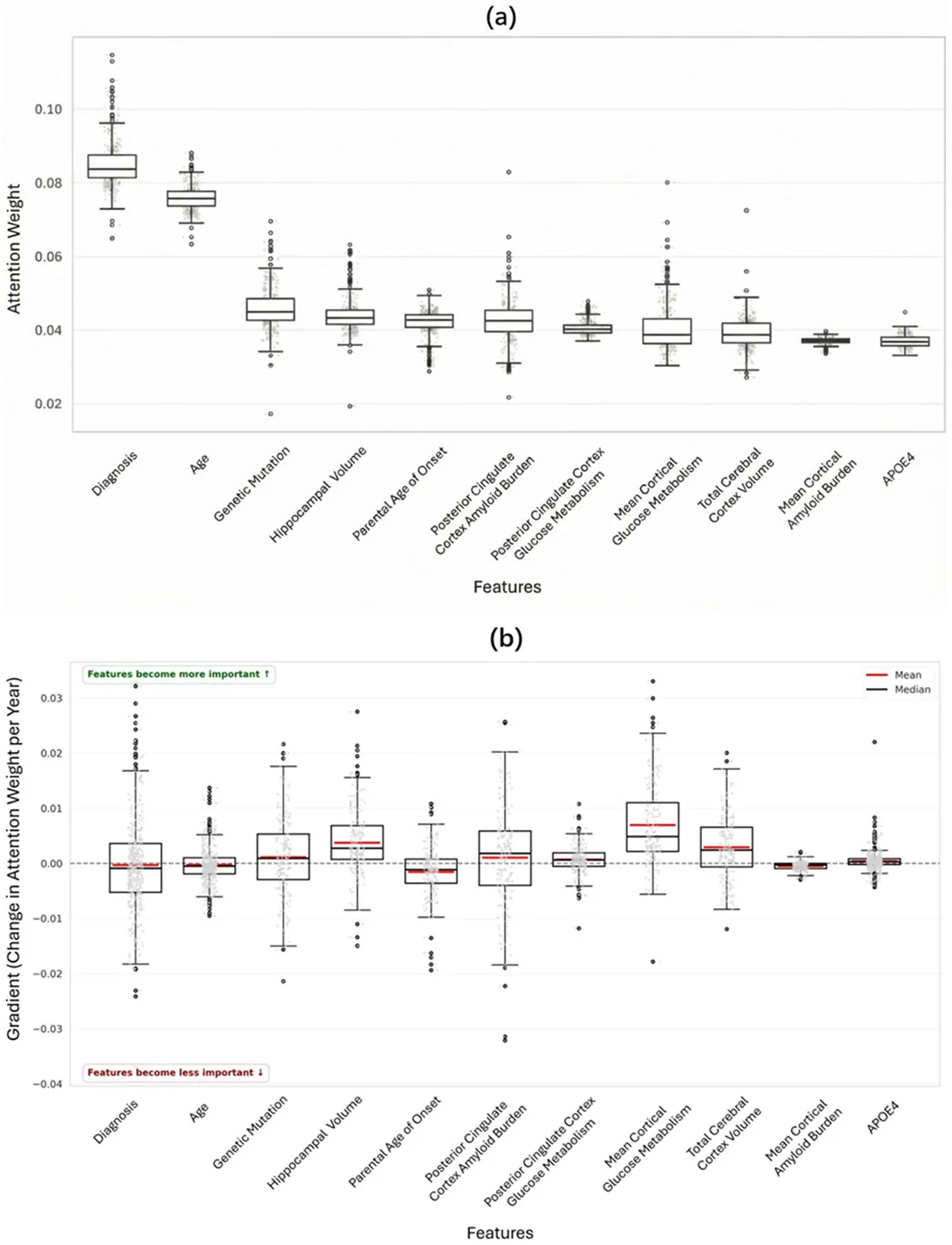
Feature attention analysis for the DIAN cohort. **(a) Distribution of Personalised Feature Attention Weights Across DIAN Cohort:** Genetic markers (Genetic Mutation, Parental Age of Onset) showed high attention weights. Neuroimaging measures (Hippocampal Volume, Posterior Cingulate Cortex Amyloid Burden, Posterior Cingulate Cortex Glucose Metabolism, Mean Cortical Glucose Metabolism, Total Cerebral Cortex Volume, and Mean Cortical Amyloid Burden) demonstrated elevated attention compared to cognitive measures in TADPOLE and ABC-DS cohorts. **(b) Temporal Gradients of Feature Importance Across DIAN Disease Progression:** Parental Age of Onset exhibited strongest negative gradient. Neuroimaging measures showed positive gradient or near-zero patterns.

Figures 3 and 4 present the distributions of feature importance and feature importance gradients for the ABC-DS and DIAN cohorts.

#### ABC-DS Adaptations:

In the absence of neuroimaging data, the model adapted by elevating attention to available clinical markers, see Figure 3a and b. APOE4 showed a high median attention weight (0.045) with a wide variance, indicating heterogeneous genetic influence. Clinical measures and daily function scores received elevated attention weights. The temporal gradient analysis showed memory and functional measures (Cued Recall Test, Premorbid Intellectual/Mental Impairment Level, and Adaptive Behaviour Composite Score from Vineland) displaying the strongest positive gradients (median gradient >0.01). Age demonstrated a stronger negative gradient in ABC-DS compared to TADPOLE patterns. This reflects the deterministic nature of AD pathology in DS, where virtually all individuals develop amyloid pathology by age 40 due to APP gene triplication, making chronological age less predictive once individuals enter the typical onset window.

Diagnosis exhibited a negative median gradient with high variance across participants. Standard diagnostic categories provided inconsistent predictive value in DS likely due to pre-existing intellectual disabilities of varying severity, which obscured clear diagnostic transitions that would typically indicate progression. The high variance reflected substantial individual differences, with some participants showing clear diagnostic progression while others were already impaired at baseline, making diagnosis unreliable as a universal predictor in this population. Karyotype showed the strongest negative gradient, reflecting its diminishing utility as a predictor once individuals enter the deterministic disease window.

#### DIAN Characteristics:

Genetic markers (Mutation) demonstrated the highest attention weights, with mutation status and parental age of onset showing median attention >0.04, see Figure 4a and b. PIB PET measures (Posterior Cingulate Cortex Amyloid Burden and Mean Cortical Amyloid Burden) displayed elevated importance (median: 0.043 and 0.038 respectively), reflecting strong amyloid pathology signatures. Several neuroimaging measures (Hippocampal Volume, Mean Cortical Glucose Metabolism and Total Cerebral Cortex Volume) showed strong positive gradients (median gradient >0.025), reflecting the importance of neuroimaging data in predicting and modelling the progression of AD in this population.

## Discussion

### 3.1 Model Performance Across Populations

Our multi-dimensional attention framework achieved substantial performance improvements over baseline models. This consistent enhancement demonstrated that attention mechanisms captured clinically meaningful patterns beyond conventional recurrent architectures, aligning with recent findings in medical time series analysis ^29^. The largest improvement in performance occurred in the DIAN cohort, where the independently trained multi-dimensional attention model achieved a 0.135 mAUC increase over the standard GRU model (0.902 vs 0.767). This improvement highlights how the attention mechanism effectively captured the predictable progression patterns created by deterministic mutations ^25^. Conversely, the model showed the smallest gain over the standard GRU in the ABC-DS dataset without transfer learning (0.680 vs 0.637). This more modest increase reflects the challenges posed by limited biomarker availability and fewer clinical visits per participant in this dataset ^19 2^.

Our framework also outperformed the Transformer and STraTS baselines across all three cohorts. Although STraTS performs strongly on general electronic health record tasks, its purely attention-based formulation appeared poorly suited to the short longitudinal sequences and heterogeneous biomarker availability of AD cohorts, particularly the smaller ABC-DS dataset. Our hybrid architecture combines recurrent temporal dynamics with multi-dimensional attention, which better reflects the sequential structure of longitudinal clinical data than purely attention-based models while also retaining interpretability.

### 3.2 Transfer Learning

#### 3.2.1 Knowledge Transfer Across Population Barriers

We observed a significant improvement in model performance in the ABC-DS cohort when the model was first trained on the TADPOLE cohort: validation mAUC improved from 0.642 to 0.779 (21.2%) and test mAUC from 0.680 to 0.771 (13.4%). This suggested our model captured fundamental disease mechanisms, such as amyloid-driven neurodegeneration, that are shared across distinct AD aetiologies. The efficacy of transfer learning for the ABC-DS cohort, which lacks the neuroimaging data of the TADPOLE training set, illustrated that the learned progression patterns are robust to variations in both disease aetiology and data modality.

Conversely, in the DIAN cohort pre-training on the TADPOLE dataset had very little impact on the model's already high predictive performance, with test mAUC remaining unchanged at 0.902 and validation mAUC showing only a marginal decrease from 0.814 to 0.810. The absence of detrimental interference here suggests that the disease progression patterns learned from the sporadic cohort are fundamentally compatible with the pathway observed in the autosomal dominant cohort. This compatibility reinforces the concept of a shared underlying neurodegenerative process, while also demonstrating that our framework can achieve high accuracy independently when comprehensive multimodal data is available.

#### 3.2.2 Biological Significance

These results align with the biological understanding that while the initial causes of AD may differ between populations (sporadic, chromosomal, or autosomal dominant), certain downstream pathological processes share common pathways ^47 48^. Our model's ability to transfer knowledge between three distinct populations provided computational evidence supporting the concept that while AD initiation mechanisms differ, the neurodegenerative cascade followed conserved pathways involving tau propagation, synaptic dysfunction, and neuronal loss.

#### 3.2.3 Clinical Utility of Transfer Learning

The performance enhancements achieved through transfer learning have direct clinical implications for populations in which comprehensive biomarker collection is constrained. For populations with DS, where comprehensive biomarker collection presents logistical and practical challenges, the proposed methodology facilitated more accurate disease progression prediction and staging despite not having access to neuroimaging data from ABC-DS. This advancement has direct relevance for clinical trial design, including better participant stratification without neuroimaging, more accurate sample size estimations, and improved identification of rapid progressors for enrichment strategies. These capabilities might also enhance longitudinal patient monitoring in specialised populations where imaging is impractical, economically expensive or burdensome for patients. Furthermore, the successful transfer learning from TADPOLE to ABC-DS demonstrates that the model can learn generalisable disease principles while retaining population-specific flexibility ^38^, suggesting potential for a universal Alzheimer’s disease progression model that adapts to available data rather than requiring full retraining for each cohort ^49^.

### 3.3 Feature Importance

#### 3.3.1 Biological Significance

The model identified three distinct biomarker tiers that emerged without a predefined hierarchy: a) anchoring features (diagnosis, age) with consistently high attention throughout disease progression; b) progression markers (cognitive/structural measures) with moderate attention and high variability reflecting individual differences; and c) risk factors (APOE4) with high baseline importance. This stratification aligned with established biology where current state indicators remained consistently important, progression markers tracked ongoing neurodegeneration ^50^, and genetic factors were crucial for determining initial disease risk but less informative for monitoring ongoing disease progression because they are static and do not change over time ^51^. The population-specific gradient signatures further validated that our model captured genuine biological heterogeneity, with each cohort displaying distinct temporal dynamics that aligned with their known pathophysiological characteristics rather than representing computational artefacts or overfitting patterns.

Population-specific patterns validated known pathophysiological differences. In ABC-DS, the prominence of APOE4 as the highly weighted feature with substantial inter-participant variance reflected the unique interaction between APOE genotype and trisomy 21, where the triplicated APP gene on chromosome 21 may modulate APOE's effects differently compared to sporadic AD. DIAN's genetic marker dominance captured the 72% variance in symptom onset explained by parental age of onset, with negative gradients reflecting diminishing importance once phenotype emerges ^20^.

Our temporal gradient analysis revealed that biological factor importance is fundamentally stage dependent. The tau biomarkers showed decreasing predictive value in late disease due to plateau effects, while cognitive measures demonstrated increasing importance as disease advanced. This stage-dependency has critical implications for clinical monitoring strategies.

#### 3.3.2 Clinical Implications of Personalised Attention Patterns

The individual-specific attention mechanisms proposed here could enable precision monitoring strategies tailored to individual disease trajectories. The temporal gradient analysis suggested that when the model identifies increasing importance for certain biomarkers over time, those participants may benefit from more frequent assessments of those markers, while stable or decreasing gradients could indicate opportunities to reduce monitoring burden. This data-driven approach might optimise clinical resources and reduce patient burden, consistent with precision medicine frameworks ^20 1^.

The negative tau gradient coupled with positive cognitive measure gradients suggested that early-stage monitoring might best prioritise CSF biomarkers ^52^, while late-stage monitoring might better emphasise cognitive assessments ^53^. This stage-dependent strategy reflects the plateau phenomenon observed in CSF total tau during advanced disease stages ^45^, where molecular biomarkers reach ceiling effects while cognitive measures continue tracking disease progression, potentially optimising both resource allocation and diagnostic accuracy throughout the disease continuum. Potential population-specific strategies also emerged from the attention patterns. TADPOLE participants might be best suited to balanced multimodal monitoring ^18^, ABC-DS participants showed high attention to APOE4 status and functional measures ^19^, and DIAN participants needed early genetic characterisation transitioning to intensive multimodal monitoring near expected onset age ^25^. Nonetheless, we note that for all datasets and most features there was high heterogeneity in feature importance between participants, potentially suggesting the importance of tailoring these strategies to specific individuals.

## Limitations

While our multi-dimensional attention framework demonstrates strong performance across diverse AD populations, several limitations warrant consideration:

### Population Representativeness:

The three cohorts, while diverse in aetiology, do not represent the full spectrum of AD. TADPOLE participants are predominantly well-educated volunteers, ABC-DS represents a specific genetic condition, and DIAN includes only rare mutation carriers. Generalisation to community-based populations requires further validation. Furthermore, while the model showed similar performance across most demographic groups, reduced accuracy was observed for Black participants (mAUC = 0.787 compared to 0.820 for White participants). This finding requires cautious interpretation given the limited sample size (N = 118) and warrants further investigation in more diverse cohorts.

### Attention Interpretability:

While our attention mechanism provides unique individual-specific insights into biomarker importance, it shares the interpretability challenges common to deep learning models ^54^. In particular, the attention mechanism cannot disambiguate between correlated features. When biomarkers are highly correlated, the model may assign high attention to one while ignoring others that are equally biologically important. For example, if hippocampal volume and entorhinal cortex thickness are correlated, the model might attend only to hippocampal volume even though both reflect the same underlying neurodegenerative process. Therefore, low attention does not imply biological irrelevance, as the feature's information may be captured through its correlated partners. High attention to a biomarker also does not specify how clinicians should act on this information. For instance, high attention to hippocampal volume tells us this feature is predictive for a given individual but does not indicate whether the participant's values are clinically concerning or what actions clinicians should take in response. Future work should focus on developing decision support frameworks that translate attention patterns into actionable clinical recommendations through collaboration with clinical experts.

### Gradient Stability:

The high variance in temporal gradients, particularly in ABC-DS and DIAN, suggests that longer follow-up periods may be needed to establish stable importance trajectories for some biomarkers.

## Conclusion

Multi-dimensional attention mechanisms successfully modelled AD progression across diverse populations with higher accuracy than conventional machine learning approaches. Importantly, pre-training on a general late-onset population sample substantially improved prediction accuracy in a more data-limited, early-onset genetic cohort, suggesting shared underlying disease mechanisms and demonstrating that knowledge from well-characterised cohorts can enhance prediction in understudied populations, including populations where neuroimaging data may not be available. Our framework also enables the generation of personalised attention patterns, revealing both universal disease principles and population-specific adaptations that reflect distinct genetic architectures. Ultimately, these results position our approach as a clinically adaptable tool that generates individual-specific hypotheses and complements clinical expertise across diverse Alzheimer's disease populations.

## Supplementary Material

This is a list of supplementary files associated with this preprint. Click to download.

• SupplementaryInformation.docx

## Figures and Tables

**Table 1:** Demographic and clinical characteristics of the three study cohorts. The Table presents data from the Alzheimer's Disease Prediction of Longitudinal Evolution (TADPOLE) dataset representing late-onset sporadic Alzheimer's disease, the Alzheimer's Biomarker Consortium - Down Syndrome (ABC-DS) dataset representing Down syndrome-associated Alzheimer's disease, and the Dominantly Inherited Alzheimer Network (DIAN) dataset representing autosomal dominant Alzheimer's disease. For each dataset, participants are stratified by diagnostic group: cognitively normal (or cognitively stable for the Down syndrome population), mild cognitive impairment (MCI), and Alzheimer's disease (AD) dementia. Available data modalities for each cohort are shown, including magnetic resonance imaging (MRI) regions of interest (ROIs) with volumetric and cortical measures, positron emission tomography (PET) imaging with fluorodeoxyglucose (FDG), florbetapir (AV45), and flortaucipir (AV1451) tracers, diffusion tensor imaging (DTI) ROIs, cerebrospinal fluid (CSF) biomarkers, apolipoprotein E (APOE) genotyping, karyotyping for chromosomal analysis, and various cognitive and neurological assessments.

Dataset						Modalities
**TADPOLE, total N = 1669**	Participant group	Number (% of all participants)	Visits per participant	Age	Gender (%female)	Demographics Diagnosis Cognitive tests MRI ROIs (volumes, cortical thicknesses, surface areas) FDG PET AV45 PET AV1451 PET DTI ROIs CSF APOE genotype
	Controls	502 (30.1%)	7.0±4.1	73.6±6.2	52.0%	
	MCI	617 (37.0%)	7.0±3.7	73.2±7.6	40.2%	
	AD dementia	550 (33.0%)	6.1±3.9	74.5±7.4	43.1%	
**ABC-DS, total N = 396**	Cognitively stable	327 (82.6%)	2.7±0.5	45±9.5	47.6%	Demographics Diagnosis Physical and neurological examinations Neuropsychological tests APOE genotype CSF Karyotyping
	MCI	29 (7.3%)	2.7±0.5	54±5.3	43.3%	
	AD dementia	40 (10.1%)	2.9±0.3	56±5.4	34.5%	
**DIAN, total N = 425**	Controls	304 (71.5%)	3.1±1.2	39.2±10.0	57.4%	Demographics Diagnosis Physical and neurological examinations Cognitive tests APOE genotype MRI PET CSF Genetic mutation
	MCI	45 (10.6%)	3.2±1.6	45.0±9.8	71.1%	
	AD dementia	76 (17.9%)	3.5±1.5	50.6±7.8	46.1%	

**Table 2.** Performance (mAUC values) of Alzheimer's disease progression prediction models across TADPOLE, ABC-DS, and DIAN datasets. Models include Support Vector Machine (SVM), eXtreme Gradient Boosting (XGBoost), Gated Recurrent Unit (GRU), Long Short-Term Memory (LSTM), Transformer, Self-supervised Transformer for Time-Series (STraTS), Multi-Dimensional Attention GRU (our proposed model), and TADPOLE-pretrained variants of the Transformer, STraTS, and Multi-Dimensional Attention GRU (transfer learning approaches). Higher mAUC values (closer to 1) indicate better performance, with 0.5 representing chance level. Bold values indicate the best performance for each dataset.

Model	TADPOLE		ABC-DS		DIAN	
	Validation	Test	Validation	Test	Validation	Test
*SVM*	0.682	0.665	0.583	0.613	0.704	0.722
*XGBoost*	0.732	0.709	0.654	0.671	0.788	0.767
*GRU*	0.771	0.677	0.629	0.637	0.783	0.767
*LSTM*	0.764	0.685	0.618	0.651	0.779	0.767
*Transformer*	0.795	0.754	0.585	0.543	0.796	0.762
*TADPOLE Pretrained Transformer*	N/A	N/A	0.689	0.665	0.812	0.826
*STraTS*	0.851	0.751	0.547	0.553	0.714	0.735
*TADPOLE Pretrained STraTS*	N/A	N/A	0.715	0.666	0.802	0.785
*Multi-Dimensional Attention GRU*	**0.856**	**0.793**	0.642	0.680	**0.814**	**0.902**
*TADPOLE Pretrained Multi-Dimensional Attention GRU*	N/A	N/A	**0.779**	**0.771**	0.810	**0.902**

## Data Availability

All data used in this study were obtained under data use agreements with the respective consortia. The TADPOLE dataset used in this study was derived from the Alzheimer's Disease Neuroimaging Initiative (ADNI) database and is publicly available at https://adni.loni.usc.edu. The ABC-DS dataset is available through the Alzheimer's Biomarker Consortium–Down Syndrome; access requires application and approval from the ABC-DS steering committee (https://www.nia.nih.gov/research/abc-ds). The DIAN dataset is available through the Dominantly Inherited Alzheimer Network; access requires application and approval from the DIAN steering committee (https://dian.wustl.edu). Python code used in this study is available upon request from the corresponding author.

## Alzheimer Biomarker Consortium- Down Syndrome (ABC-DS) Investigators

Beau M. Ances, MD PhD; Howard F. Andrews, PhD; Karen Bell, MD; Rasmus M. Birn, PhD; Adam M. Brickman, PhD; Peter Bulova, MD; Jeff Burns, MD; Amrita Cheema, PhD; Kewei Chen, PhD; Bradley T. Christian, PhD; Isabel Clare, PhD; Ann D. Cohen, PhD; Eric W. Doran, MS; Tatiana M. Foroud, PhD; Benjamin L. Handen, PhD; Jordan Harp, PhD; Sigan L. Hartley, PhD; Elizabeth Head, PhD; Denise Head, PhD; Christy Hom, PhD; Lawrence Honig, MD; Milos D. Ikonomovic, MD; Sterling C Johnson, PhD; M. Ilyas Kamboh, PhD; David Keator, PhD; Julia K. Kofler, MD; William Charles Kreisl, MD; Sharon J. Krinsky-McHale, PhD; Florence Lai, MD; Patrick Lao, PhD; Charles Laymon, PhD; Joseph Hyungwoo Lee, PhD; Ira T. Lott, MD; Mark Mapstone, PhD; Davneet Singh Minhas, PhD; Neelesh Nadkarni, MD; Sid O’Bryant, PhD; Deborah Pang, MPH; Melissa Petersen, PhD; Julie C. Price, PhD; Lauren Ptomey, PhD; Margaret Pulsifer, PhD; Michael S. Rafii, MD PhD; Herminia Diana Rosas, MD; Frederick Schmitt, PhD; Nicole Schupf, PhD; Wayne P. Silverman, PhD; Dana L. Tudorascu, PhD; Rameshwari Tumuluru, MD; Badri Varadarajan, PhD; Michael A. Yassa, PhD; Shahid Zaman, MD PhD; Fan Zhang, PhD

## Dominantly Inherited Alzheimer Network (DIAN) Investigators

Randall Bateman; Alisha J. Daniels; Laura Courtney; Angela Ziegemeier; Karina Skrbec; Cortaiga Hellm; Mariana Martin; Ellen Ziegemeier; Jamie Bartzel; Eric McDade; Jorge J. Llibre-Guerra; Charlene Supnet-Bell; Chengie Xiong; Xiong Xu; Ruijin Lu; Guoqiao Wang; Yan Li; Yuzheng Nie; Emily Gremminger; Jyoti Arora; Richard J. Perrin; Erin E. Franklin; Laura Ibanez; Gina Jerome; Jennifer Stauber; Bryce Baker; Matthew Minton; Sam Preminger; Carlos Cruchaga; Alison M. Goate; Alan E. Renton; Danielle M. Picarello; Brian Fulton-Howard; Tammie L.S. Benzinger; Brian A. Gordon; Jessica Banks; Russ Hornbeck; Allison Chen; Charles Chen; Shaney Flores; Manu Goyal; Nelly Joseph-Mathurin; Kelley Jackson; Sarah Keefe; Deborah Koudelis; Parinaz Massoumzadeh; Nicole McKay; Qing Wang; Edita Sabaredzovic; Jalen Scott; Ashlee Simmons; Jacqueline Rizzo; Andrei Vlassenko; Yong Wang; Thomas Smith; Mei Murphy; Beau Ances; Kaitlyn Dombrowski; David Hoagey; Peter Millar; Savannah Powles; Griffin Melson; Jason Hassenstab; Jennifer Smith; Sarah Stout; Clara Vila-Castelar; Colleen Frank; Andrew J. Aschenbrenner; Celeste M. Karch; Jacob Marsh; John C. Morris; David M. Holtzman; Nicolas R. Barthélemy; Jinbin Xu; Sarah B. Berman; Neelesh Nadkarni; Snezana Ikonomovic; Gregory S. Day; Christian Lachner; Martin Farlow; Jasmeer P. Chhatwal; Valentina Pinilla; Courtney Maa; Takeshi Ikeuchi; Takanobu Ishiguro; Azusa Aoyama; Kenji Ishii; Michio Senda; Yoshiki Niimi; Edward D. Huey; Courtney Bodge; Stephen Salloway; Emma Devenney; Peter R. Schofield; William S. Brooks; Jacob A. Bechara; Ralph N. Martins; Hamid R. Sohrabi; Kevin Taddei; Samantha L. Gardener; Nick C. Fox; David M. Cash; Natalie S. Ryan; Mathias Jucker; Christoph Laske; Oksana Forkavets; Beatrice Spring; Susanne Graber-Sultan; Christian la Fougère; Gerald Reischl; Ulrike Obermueller; Johannes Levin; Ilona Rude; Jonathan Vöglein; Jae-Hong Lee; Jee Hoon Roh; Ricardo F. Allegri; Patricio Chrem Mendez; Ezequiel Surace; Ana Sosa Ortiz; Gabriela Vigo; David Aguillon; Alejandro Guerrero; Yudy Milena Leon; Laura Ramirez; Laura Serna; Yamile Bocanegra; Suman Jayadev; Allan I. Levey; Erik C.B Johnson; Nicholas T. Seyfried; John Ringman; Anne M. Fagan; Hiroshi Mori; Colin Masters; James M. Noble; Raquel Sanchez-Valle; Francisco Lopera

## List of abbreviations

ABC-DS: Alzheimer's Biomarker Consortium – Down Syndrome
AD: Alzheimer's disease
ADAS11: Alzheimer's Disease Assessment Scale – Cognitive Subscale, 11 items
ADAS13: Alzheimer's Disease Assessment Scale – Cognitive Subscale, 13 items
ADNI: Alzheimer's Disease Neuroimaging Initiative
APOE: Apolipoprotein E
APP: Amyloid Precursor Protein
AUC: Area Under the Receiver Operating Characteristic Curve
AV45: Florbetapir (PET tracer)
AV1451: Flortaucipir (PET tracer)
CN: Cognitively Normal
CS: Cognitively Stable
CSF: Cerebrospinal Fluid
DIAN: Dominantly Inherited Alzheimer Network
DS: Down Syndrome
DTI: Diffusion Tensor Imaging
FAQ: Functional Activities Questionnaire
FDG: Fluorodeoxyglucose
GRU: Gated Recurrent Unit
HCRW: Health and Care Research Wales
HRA: Health Research Authority
IRAS: Integrated Research Application System
KNN: K-Nearest Neighbours
LSTM: Long Short-Term Memory
mAUC: Multi-class Area Under the Receiver Operating Characteristic Curve
MCI: Mild Cognitive Impairment
MRI: Magnetic Resonance Imaging
NHS: National Health Service
PET: Positron Emission Tomography
PIB: Pittsburgh Compound B
PSEN1: Presenilin 1
PSEN2: Presenilin 2
RAVLT: Rey Auditory Verbal Learning Test
REC: Research Ethics Committee
ReLU: Rectified Linear Unit
RETAIN: Reverse Time Attention (model)
RNN: Recurrent Neural Network
ROI: Region of Interest
STraTS: Self-supervised Transformer for Time-Series
SVM: Support Vector Machine
TADPOLE: The Alzheimer's Disease Prediction Of Longitudinal Evolution
tanh: Hyperbolic Tangent
XGBoost: eXtreme Gradient Boosting

## References

[1] Scheltens P. Alzheimer’s disease. Lancet 397, 1577–1590 (2021). 33667416 10.1016/S0140-6736(20)32205-4 PMC8354300

[2] Fortea J. Clinical and biomarker changes of Alzheimer’s disease in adults with Down syndrome: a cross-sectional study. Lancet 395, 1988–1997 (2020). 32593336 10.1016/S0140-6736(20)30689-9 PMC7322523

[3] Bateman R. J. Clinical and Biomarker Changes in Dominantly Inherited Alzheimer’s Disease. N. Engl. J. Med. 367, 795–804 (2012). 22784036 10.1056/NEJMoa1202753 PMC3474597

[4] Aisen P. S., Jimenez-Maggiora G. A., Rafii M. S., Walter S. & Raman R. Early-stage Alzheimer disease: getting trial-ready. Nat. Rev. Neurol. 18, 389–399 (2022). 35379951 10.1038/s41582-022-00645-6 PMC8978175

[5] Petersen R. C. Alzheimer’s Disease Neuroimaging Initiative (ADNI): Clinical characterization. Neurology 74, 201–209 (2010). 20042704 10.1212/WNL.0b013e3181cb3e25 PMC2809036

[6] Arya A. D. A systematic review on machine learning and deep learning techniques in the effective diagnosis of Alzheimer’s disease. Brain Informatics 10, (2023). 10.1186/s40708-023-00195-7 PMC10349019 37450224

[7] Malik I., Iqbal A., Gu Y. H. & Al-antari M. A. Deep Learning for Alzheimer’s Disease Prediction: A Comprehensive Review. Diagnostics 14, 1–23 (2024). 10.3390/diagnostics14121281 PMC11202897 38928696

[8] Khojaste-sarakhsi M., Shahabi S., Ghomi S. M. T. F. & Marchiori E. Deep learning for Alzheimer ’ s disease diagnosis : A survey. Artif. Intell. Med. 130, 102332 (2022). 35809971 10.1016/j.artmed.2022.102332

[9] Ebrahimighahnavieh A., Luo S. & Chiong R. Computer Methods and Programs in Biomedicine Deep learning to detect Alzheimer ’ s disease from neuroimaging : A systematic literature review. Comput. Methods Programs Biomed. 187, 105242 (2020). 31837630 10.1016/j.cmpb.2019.105242

[10] Mubonanyikuzo V. Detection of Alzheimer Disease in Neuroimages Using Vision Transformers: Systematic Review and Meta-Analysis. J. Med. Internet Res. 27, (2025). 10.2196/62647 PMC11840381 39908541

[11] Morid M. A., Sheng O. R. L. & Dunbar J. Time Series Prediction Using Deep Learning Methods in Healthcare. ACM Trans. Manag. Inf. Syst. 14, (2023).

[12] Hochreiter S. & Schmidhuber J. Long Short-Term Memory. Neural Comput. 9, 1735–1780 (1997). 9377276 10.1162/neco.1997.9.8.1735

[13] Choi E. RETAIN: An interpretable predictive model for healthcare using reverse time attention mechanism. Adv. Neural Inf. Process. Syst. 3512–3520 (2016).

[14] Vaswani A. Attention Is All You Need. (2017).

[15] Tipirneni S., Reddy C. K. & Tech V. Self-Supervised Transformer for Sparse and Irregularly Sampled Multivariate Clinical Time-Series. 16, (2022).

[16] Sun Q., Xie N., Tang B., Li R. & Shen Y. Alzheimer’s disease: From genetic variants to the distinct pathological mechanisms. Front. Mol. Neurosci. 10, 1–14 (2017). 29056900 10.3389/fnmol.2017.00319 PMC5635057

[17] Valdes P. Integrative multiomics reveals common endotypes across PSEN1, PSEN2, and APP mutations in familial Alzheimer’s disease. Alzheimer’s Res. Ther. 17, (2025). 10.1186/s13195-024-01659-6 PMC11699654 39754192

[18] Marinescu R. V. The Alzheimer’s Disease Prediction Of Longitudinal Evolution (TADPOLE) Challenge: Results after 1 Year Follow-up. Mach. Learn. Biomed. Imaging 1, 1–60 (2021).

[19] Handen B. L. The Alzheimer’s Biomarker Consortium-Down Syndrome: Rationale and methodology. Alzheimer’s Dement. Diagnosis, Assess. Dis. Monit. 12, 1–15 (2020). 10.1002/dad2.12065 PMC7396809 32775597

[20] McDade E. Longitudinal cognitive and biomarker changes in dominantly inherited Alzheimer disease. Neurology 91, E1295–E1306 (2018). 30217935 10.1212/WNL.0000000000006277 PMC6177272

[21] Schworer E. K. Lifestyle Composite and Resilience to Alzheimer ’ s Disease Pathology in Down Syndrome. 1–11 (2025) doi:10.1111/jar.70109. PMC12344623 40799171

[22] Zammit M. D. Characterizing the emergence of amyloid and tau burden in Down syndrome. 388–398 (2024) doi:10.1002/alz.13444. PMC10843570 37641577

[23] Chen H. Transferability of Alzheimer’s disease progression subtypes to an independent population cohort. Neuroimage 271, 120005 (2023). 36907283 10.1016/j.neuroimage.2023.120005

[24] Handen B. L. The Alzheimer’s Biomarker Consortium–Down Syndrome (ABC-DS): A 10-year report. Alzheimer’s Dement. 21, 1–15 (2025). 10.1002/alz.70294 PMC12079517 40371686

[25] McKay N. S. Positron emission tomography and magnetic resonance imaging methods and datasets within the Dominantly Inherited Alzheimer Network (DIAN). Nat. Neurosci. 26, 1449–1460 (2023). 37429916 10.1038/s41593-023-01359-8 PMC10400428

[26] Heinrichs B. & Eickhoff S. B. Your evidence ? Machine learning algorithms for medical diagnosis and prediction. 1435–1444 (2020) doi:10.1002/hbm.24886. PMC7268052 31804003

[27] Niu Z., Zhong G. & Yu H. A review on the attention mechanism of deep learning. Neurocomputing 452, 48–62 (2021).

[28] Murti D. M. P., Pujianto U., Wibawa A. P. & Akbar M. I. K-Nearest Neighbor (K-NN) based Missing Data Imputation. Proceeding - 2019 5th Int. Conf. Sci. Inf. Technol. Embrac. Ind. 4.0 Towar. Innov. Cyber Phys. Syst. ICSITech 2019 83–88 (2019) doi:10.1109/ICSITech46713.2019.8987530.

[29] Olaimat M. Al. TA-RNN : an attention-based time-aware recurrent neural. 169–179 (2024). 10.1093/bioinformatics/btae264 PMC11211851 38940180

[30] Adachi S. Sigsoftmax : Reanalysis of the Softmax Bottleneck. 0–11 (2026) doi:10.5555/3326943.3326970.

[31] Golovanevsky M., Eickhoff C. & Singh R. Research and Applications Multimodal attention-based deep learning for Alzheimer ’ s disease diagnosis. 29, 2014–2022 (2022). 10.1093/jamia/ocac168 PMC9667156 36149257

[32] Lau M. M. & Lim K. H. Review of adaptive activation function in deep neural network. 2018 IEEE EMBS Conf. Biomed. Eng. Sci. IECBES 2018 - Proc. 686–690 (2019) doi:10.1109/IECBES.2018.08626714.

[33] Ba J. L., Kiros J. R. & Hinton G. E. Layer Normalization. (2016).

[34] Xiong R. On layer normalization in the transformer architecture. 37th Int. Conf. Mach. Learn. ICML 2020 PartF16814, 10455–10464 (2020).

[35] Liu C., Wei Z., Zhou L. & Shao Y. Multidimensional time series classification with multiple attention mechanism. Complex Intell. Syst. 11, 1–15 (2025).

[36] Che Z., Purushotham S., Cho K., Sontag D. & Liu Y. Recurrent Neural Networks for Multivariate Time Series with Missing Values. Sci. Rep. 8, 1–12 (2018). 29666385 10.1038/s41598-018-24271-9 PMC5904216

[37] Cho K. Learning phrase representations using RNN encoder-decoder for statistical machine translation. EMNLP 2014 - 2014 Conf. Empir. Methods Nat. Lang. Process. Proc. Conf. 1724–1734 (2014) doi:10.3115/v1/d14-1179.

[38] Qiu S. Multimodal deep learning for Alzheimer’s disease dementia assessment. Nat. Commun. 13, 1–17 (2022). 35725739 10.1038/s41467-022-31037-5 PMC9209452

[39] Oh K., Chung Y. C., Kim K. W., Kim W. S. & Oh I. S. Classification and Visualization of Alzheimer’s Disease using Volumetric Convolutional Neural Network and Transfer Learning. Sci. Rep. 9, 1–16 (2019). 31796817 10.1038/s41598-019-54548-6 PMC6890708

[40] Bartlett C. W. Invasive or More Direct Measurements Can Provide an Objective Early-Stopping Ceiling for Training Deep Neural Networks on Non-invasive or Less-Direct Biomedical Data. SN Comput. Sci. 4, 1–12 (2023). 10.1007/s42979-022-01553-8 PMC9836982 36647373

[41] Montavon G. Neural Networks: Tricks of the Trade. Springer Lecture Notes in Computer Sciences (2012).

[42] Nakamura K., Derbel B., Won K. J. & Hong B. W. Learning-rate annealing methods for deep neural networks. Electron. 10, 1–12 (2021).

[43] Yang L. & Wu T. T. Model-based clustering of high-dimensional longitudinal data via regularization. Biometrics 79, 761–774 (2023). 35428983 10.1111/biom.13672

[44] Chen T. & Guestrin C. XGBoost : A Scalable Tree Boosting System. 785–794 (2016).

[45] Williams J. H. Non-linear relationships of cerebrospinal fluid biomarker levels with cognitive function: An observational study. Alzheimer’s Res. Ther. 3, 5 (2011). 21329517 10.1186/alzrt64 PMC3109414

[46] Sutphen C. L. Longitudinal decreases in multiple cerebrospinal fluid biomarkers of neuronal injury in symptomatic late onset Alzheimer’s disease. Alzheimer’s Dement. 14, 869–879 (2018). 29580670 10.1016/j.jalz.2018.01.012 PMC6110083

[47] Gan L., Cookson M. R., Petrucelli L. & La Spada A. R. Converging pathways in neurodegeneration, from genetics to mechanisms. Nat. Neurosci. 21, 1300–1309 (2018). 30258237 10.1038/s41593-018-0237-7 PMC6278826

[48] Hampel H. The Amyloid-β Pathway in Alzheimer’s Disease. Mol. Psychiatry 26, 5481–5503 (2021). 34456336 10.1038/s41380-021-01249-0 PMC8758495

[49] Venugopalan J., Tong L., Hassanzadeh H. R. & Wang M. D. Multimodal deep learning models for early detection of Alzheimer’s disease stage. Sci. Rep. 11, 1–13 (2021). 33547343 10.1038/s41598-020-74399-w PMC7864942

[50] Jack C. R. NIA-AA Research Framework: Toward a biological definition of Alzheimer’s disease. Alzheimer’s Dement. 14, 535–562 (2018). 29653606 10.1016/j.jalz.2018.02.018 PMC5958625

[51] Belloy M. E., Napolioni V. & Greicius M. D. A Quarter Century of APOE and Alzheimer’s Disease: Progress to Date and the Path Forward. Neuron 101, 820–838 (2019). 30844401 10.1016/j.neuron.2019.01.056 PMC6407643

[52] Hansson O. The Alzheimer’s Association appropriate use recommendations for blood biomarkers in Alzheimer’s disease. Alzheimer’s Dement. 18, 2669–2686 (2022). 35908251 10.1002/alz.12756 PMC10087669

[53] Jutten R. J. Finding Treatment Effects in Alzheimer Trials in the Face of Disease Progression Heterogeneity. Neurology 96, E2673–E2684 (2021). 34550903 10.1212/WNL.0000000000012022 PMC8205463

[54] Kim J. Limitations of deep learning attention mechanisms in clinical research: Empirical case study based on the Korean diabetic disease setting. J. Med. Internet Res. 22, 1–19 (2020). 10.2196/18418 PMC7773508 33325832

